# Quick Optical Identification of the Defect Formation in Monolayer WSe_2_ for Growth Optimization

**DOI:** 10.1186/s11671-019-3110-z

**Published:** 2019-08-14

**Authors:** Long Fang, Haitao Chen, Xiaoming Yuan, Han Huang, Gen Chen, Lin Li, Junnan Ding, Jun He, Shaohua Tao

**Affiliations:** 1 Hunan Key Laboratory of Super Micro-structure and Ultrafast Process, School of Physics and Electronics, Central South University, Changsha, 410083 China; 2 College of Advanced Interdisciplinary Studies, National University of Defense Technology, Changsha, 410083 China; 3 School of Materials Science and Engineering, Central South University, Changsha, 410083 China

**Keywords:** WSe_2_, Defects, Crystal stability, Photoluminescence, Raman scattering

## Abstract

Bottom-up epitaxy has been widely applied for transition metal dichalcogenides (TMDCs) growth. However, this method usually leads to a high density of defects in the crystal, which limits its optoelectronic performance. Here, we show the effect of growth temperature on the defect formation, optical performance, and crystal stability in monolayer WSe_2_ via a combination of Raman and photoluminescence (PL) spectroscopy study. We found that the defect formation and distribution in monolayer WSe_2_ are closely related to the growth temperature. These defect density and distribution can be controlled by adjusting the growth temperature. Aging experiments directly demonstrate that these defects are an active center for the decomposition process. Instead, monolayer WSe_2_ grown under optimal conditions shows a strong and uniform emission dominated by neutral exciton at room temperature. The results provide an effective approach to optimize TMDCs growth.

**Electronic supplementary material:**

The online version of this article (10.1186/s11671-019-3110-z) contains supplementary material, which is available to authorized users.

## Introduction

Ultrathin TMDCs (MX_2_, M = Mo, W; X = Se, S, etc.) have been widely applied in the photonic and optoelectronic application fields, such as photodetectors [1–4], ultrathin transistors [5, 6], photovoltaic devices [7, 8], sensors [9, 10], and electrocatalysis [11]. Compared with mechanical exfoliation method, chemical vapor deposition (CVD) shows great advantages in massive production, morphology, and structure controlling [12–15], which are highly desired for large-area flexible material development and optoelectronic device applications [2, 16–18]. However, the formation of lattice defects in two-dimensional (2D) materials during the CVD growth is detrimental to its photoelectric properties, device performance, and even the crystal stability. For example, the hole mobility of WSe_2_ field-effect transistor fabricated using CVD grown monolayer is far below the theoretical predictions [19]. The defect formation-induced nonuniform photoluminescence (PL) emission distribution has been widely observed in the grown TMDCs monolayer [20–24]. CVD-grown TMDCs monolayer shows poor lattice stability in the air [25]. The high defect density in CVD-grown 2D materials significantly limits their device performance and stability, especially for devices exposed to the air for a long time.

The most direct and effective methods for 2D materials defect detection are transmission electron microscopy (TEM) [26] and scanning tunneling microscopy (STM) technique [27]. But these methods usually require sample transferring which could cause new defects. In addition, these methods are time-consuming and only detect the defects in a small area. For the growth optimization, a quick and nondestructive evaluation method is highly demanded. Raman spectroscopy is an important and nondestructive method to probe the lattice vibration, lattice distortion, and electronic properties of materials [28, 29]. For instance, the XeF_2_ treatment-induced defects in WSe_2_ have been studied by comparing the E^1^_2g_ peak intensity, the peak shift, and the full width at half maximum (FWHM) [30]. PL spectroscopy shows advantages in quickly determining the optical properties and detecting the electronic structure TMDCs without damaging. So it is widely used to study the optical properties of TMDCs [2, 31, 32]. In addition, PL is quite sensitive to the excitons, trions, and defects in monolayer TMDCs [33–36]. Rosenberger et al. show an inverse relationship between the PL intensity of monolayer WS_2_ and defect density [21]. Further research shows that the weak PL is mostly due to the formation of negatively charged excitons [37]. Therefore, optical characterization offers a quick and nondestructive method to evaluate the localized defects and crystal quality of TMDCs.

Growth time and growth temperature are the two most important parameters affecting the growth of 2D materials. These effects on the growth duration of CVD-grown WSe_2_ monolayer have been reported before [38]. Therefore, in this work, we try to focus on the optical property difference of WSe_2_ grown at different temperatures and study the defect-induced crystal stability differences. The optical performance and the lattice quality are examined using confocal Raman and PL techniques for growth optimization. The crystal defects are found to weaken the PL emission intensity and lead to a nonuniform emission distribution in the triangle WSe_2_ domain due to defect density difference. Moreover, these defects cause a low energy emission peak in the PL spectrum, as observed in both room temperature and low-temperature PL spectra. In addition to the negative effect on the optical performance, the defects deteriorate the crystal stability in the air, resulting in faster decomposition rate of WSe_2_. Based on the optical characterization results, we found that there exists an optical growth temperature for WSe_2_. In our case, this temperature is 920 °C. Either reducing or increasing the growth temperature impacts the optical properties and crystal stability of monolayer WSe_2_. These results provide an approach for us to optimize the optical properties and crystal stability of 2D materials [39].

## Methods

### Synthesis of Monolayer WSe_2_

Monolayer WSe_2_ was synthesized using high-purity Se powder (Alfa-Aesar 99.999%) and WO_3_ powder (Aladdin 99.99%) using a 2-inch-diameter quartz tube furnace. The Se powders (30 mg) were placed in a quartz boat at the first heating zone. WO_3_ powders (100 mg) were placed in a quartz boat at the second heating zone. The distance between the Se powder and WO_3_ powder is about 25 cm. *c*-plane (0001) sapphire substrates were cleaned and placed at downstream (5~10 cm) of the WO_3_ solid sources. Before the experiments, the chamber was pumped about 10 min and flushed with high-purity Ar carrier gas (99.9999 %) under a flow of 200 standard-state cubic centimeter per minute (sccm) at room temperature to remove the oxygen contamination. After that, 10% H_2_ and Ar mixture gas with a flow of 50 sccm was introduced into the furnace at an ambient pressure. The second heating zone was heated to the target temperature (860~940 °C) at a ramping rate of 20 °C/min. After that, the temperature was maintained at the growth temperature for 6 min. Meanwhile, the first heating zone was kept at 320 °C. After growth, the furnace was cooled to room temperature.

### Characterization

The morphology of as-grown WSe_2_ was examined using an optical microscopy (NPLANEPi100X). Raman scattering and micro-PL measurements were performed using a Renishaw system (inVia Qontor). The excitation was pumped through an objective lens (× 100) with a green (532 nm) laser and 1800 lines/mm grating. Atomic force microscope (AFM) measurements were performed using an Agilent system (Agilent 5500, Digital Instruments, tapping mode). The morphology changes of monolayer WSe_2_ were examined by scanning electron microscopy (SEM, TESCAN MIRA3 LMU).

## Results and Discussion

**Fig. 1:**
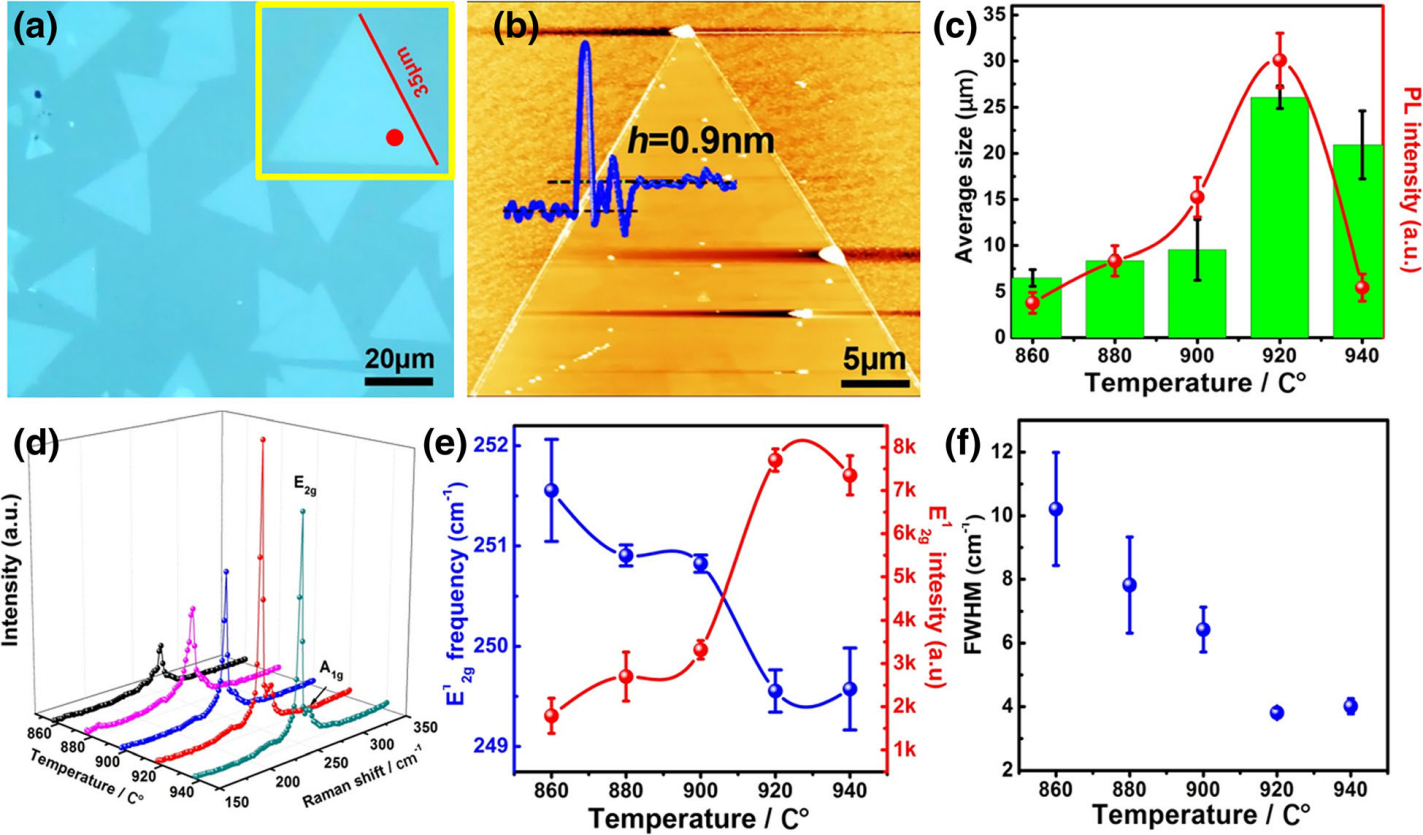
The growth optimization of monolayer WSe_2_ on sapphire substrate. **a** Optical and **b** the corresponding AFM images of triangular monolayer WSe_2_ grown at 920 °C. **c** The average domain size and integrated PL intensity. **d** Raman spectra. **e** The E^1^_2g_ frequency and intensity together with **f** FWHM of E^1^_2g_ peak for monolayer WSe_2_ grown from 860 °C to 940 °C. All the Raman and PL spectra were taken from the similar region from the triangle monolayer WSe_2_, as pointed out by a red point in **a**

The effect of growth temperature on the WSe_2_ was performed during the temperature range from 860 to 940 °C. Statistical analysis of optical microscopy images and PL performance indicates that the optimal growth temperature is 920 °C, as demonstrated in Fig. 1a, c. Furthermore, at 920 °C, the effect of growth time on the sizes and density of CVD-grown WSe_2_ flakes has been studied. The size of WSe_2_ flakes gradually increases with time (3–20 min), and the obtained results are quite similar to those published before [38]. When the growth time is 20 min, even a millimeter-scale WSe_2_ film can be grown. After film formation, a second layer is formed (more optical microscopy images and PL statistics are shown in Additional file 1: Figure S1–S3 in the supporting information (SI)). Under the 920 °C, a high density of triangular WSe_2_ domain with uniform size is formed with an average edge length of ~ 35 μm. AFM characterization shows a thickness of ~ 0.9 nm (see Fig. 1b). Furthermore, the Raman scattering detects the characteristic vibration modes (E^1^_2g_ and A_1g_) of WSe_2_ to be at ~ 249.5 and ~ 260 cm^−1^, respectively (see Fig. 1d), which have also been observed in previous reports [38, 40]. No B_2g_ (308 cm^−1^) mode which represents the vibration between different layers is detected [30, 41]. These results indicate that the as-grown WSe_2_ is monolayer. Lowering or increasing the growth temperature leads to a drop of both the density and size of WSe_2_ domains. At low growth temperature (860 °C), the density of WSe_2_ is much lower and the grain size is reduced to ~ 5 μm. Improving the growth temperature to 920 °C increases the nucleation density and the crystal growth speed (see Fig. 1c) [42]. The domain size drops again as the temperature exceeds 920 °C, which is probably due to a higher decomposition velocity. Despite the morphology difference, the grown WSe_2_ during the investigated temperature range (860 to 940 °C) are all monolayer. The photon emission intensity and the domain size evolution trend with temperature are quite similar, posing the strongest PL emission intensity at 920 °C (see Fig. 1c). This emission intensity difference suggests that even though monolayer WSe_2_ can be obtained under different growth temperatures, however, their optical performance varies drastically. The reason for this PL emission difference can be revealed by the Raman scattering as well. Figure 1d compares the Raman spectra of WSe_2_ at different growth temperature, from 860 to 940 °C (more Raman spectroscopy statistics are shown in Additional file 1: Figure S4). The absence of B_2g_ mode indicates that WSe_2_ is monolayer grown at different temperatures [30, 41]. The E^1^_2g_ frequency and intensity are related to the strain level and crystal quality [23, 43, 44], and the FWHM of the Raman peak can reflect the 2D materials crystal quality. The narrower FWHM indicates a higher crystal quality of the 2D materials [12]. Both experiments and theoretic calculations demonstrate that E^1^_2g_ peaks around 249.5 cm^−1^ for ideal WSe_2_ monolayer crystal [41, 45]. Figure 1e shows the E^1^_2g_ frequency and intensity as a function of temperature. The E^1^_2g_ frequency drops from 251.5 cm^−1^ to a minimum of 249.5 cm^−1^ at 920 °C before increases again during the investigated temperature range, and the FWHM shows a similar trend as the E^1^_2g_ frequency (see Fig. 1f). In addition, the E^1^_2g_ peak intensity poses a maximum intensity at 920 °C. Considering the highest Raman scattering intensity, the narrowest FWHM, the perfect matched Raman peak (the E^1^_2g_ peak is around 249.5 cm^−1^ for ideal monolayer WSe_2_), and the strongest PL emission intensity, we demonstrate that monolayer WSe_2_ grown at 920 °C shows the purist crystal quality [12, 30].

**Fig. 2:**
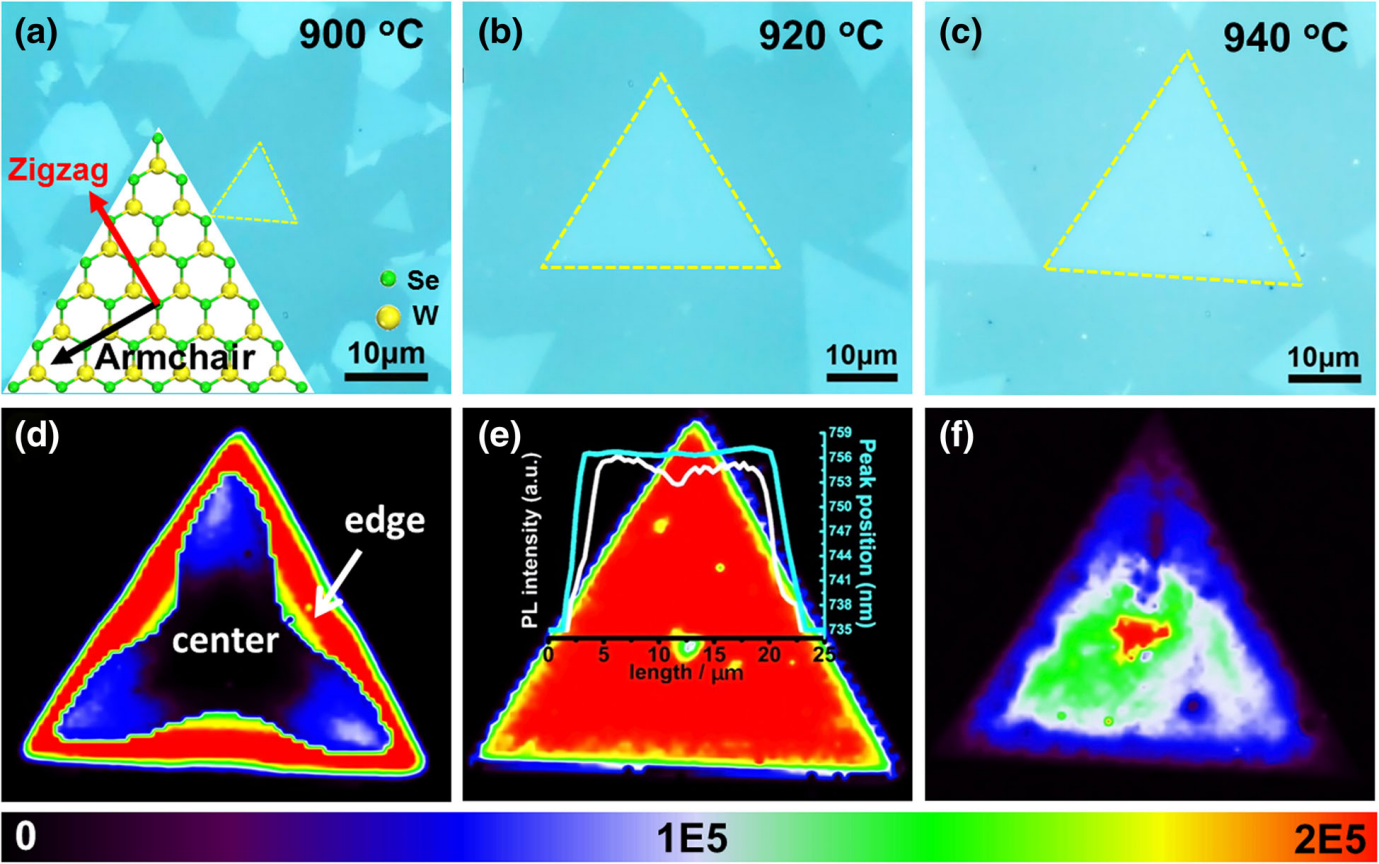
PL integral (range 725–785 nm) mapping of the monolayer WSe_2_ grown under different temperatures together with the corresponding optical images. **a**, **d** 900 °C. **b**, **e** 920 °C. **c**, **f** 940 °C. The inset in **a** is an atomic illustration of the WSe_2_ layer showing the armchair direction. The excitation power for the PL mapping is 50 μW

**Fig. 3:**
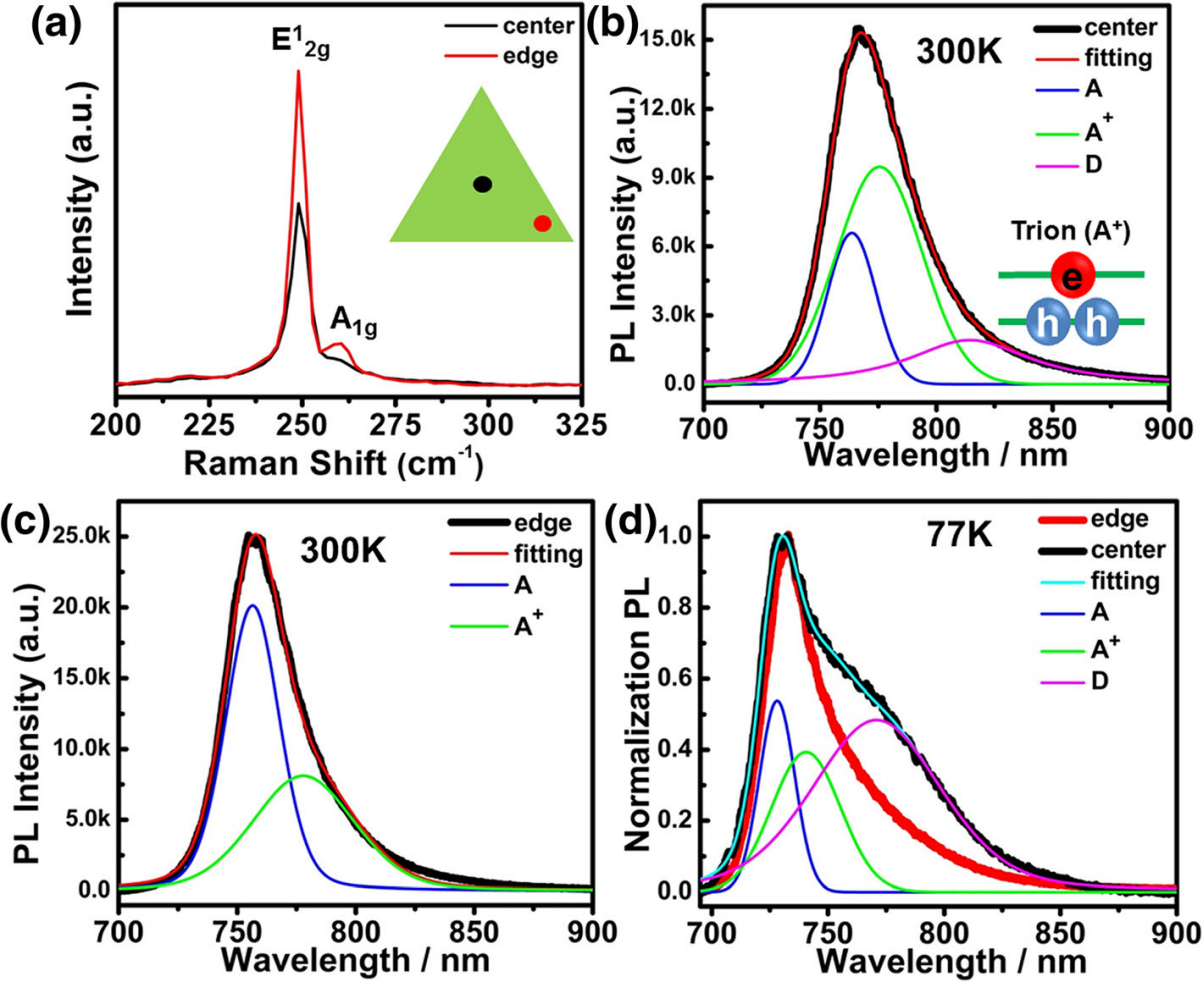
**a** Raman spectra obtained from the center region and edge region at 50 μW excitation laser power levels. PL spectra confirm the existence of crystal defects in WSe_2_ grown at 900 °C. Room temperature PL spectra from the **b** center and **c** edge of the WSe_2_ together with fitted spectra using voigt (50% Gaussian, 50% Lorentzian) equation. **d** Low temperature (77 K) PL spectra from the center position and the edge position showing a strong defect-related peak from the center region. The PL spectrum at 77 K from the center region is fitted with three peaks

The emission intensity uniformity of the grown WSe_2_ monolayer is examined by PL mapping, as compared in Fig. 2, showing a temperature-dependent emission intensity distribution. The photon emission of WSe_2_ layer grown at 920 °C distributes uniformly for the entire monolayer except for the center region where WO_*3-x*_ and WO_3-*x*_Se_*y*_ are formed under Se-deficient atmosphere as a nucleation center for the continued WSe_2_ growth [46–48]. The inset PL intensity line scanning results further confirm the constant emission intensity and the emission energy. However, the PL emission intensity turns to inhomogeneous for other growth temperatures (see Fig. 2d–f). For lower growth temperature (900 °C), the emission intensity from the inner concave triangle region is much weaker than those close to the triangle edge. According to the WSe_2_ atom arrangement in a triangle domain [49, 50], the weak emission is along the armchair direction. Under a higher growth temperature (940 °C, see Fig. 2f), the PL intensity map poses another intensity pattern. The strongest PL intensity occurs at the center area and progressively decreases to the triangle edge (see more examples in Additional file 1: Figure S5). This emission difference cannot be observed by optical or AFM measurements. PL emission in monolayer TMDCs crystal is usually nonuniform and has been observed quite a few times in both CVD-grown [21–23, 51–53] and mechanically exfoliated layers [24, 54–56]. The main causes of nonuniform PL emission include lattice defects (including impurities [56, 57] and vacancies [27]), localized electronic states [52, 58], strain [43], and edge effect [22]. In our experiment, no similar feature due to localized electronic states or edge effect is observed. The strain should not be the main factor causing the distribution of PL intensity due to the following reasons. First, for WSe_2_ grown at 900 °C, the center and the edge regions undergo the same heat treatment; the resulted strain level should be the same [59]. Secondly, Kim et al. compared the PL of WS_2_ before and after transferring to transmission electron microscopy (TEM) copper grid, excluding the possibility of the substrate caused nonuniform PL and Raman distribution [58]. Thirdly, the E^1^_2g_ mode is sensitive to the strain and is used to estimate the strain level [44]. The E^1^_2g_ peak of center and edge region in monolayer WSe_2_ growth at 900 °C is the same (249 cm^−1^) without any sign of peak shift (as shown in Fig. 3a), indicating a nearly constant strain level distribution between the substrate and WSe_2_. According to the above discussions, we speculate that the emission inhomogeneous is a reflection of the defect density distribution. The emission intensity from the bright emission region of samples grown at different temperatures is quite similar, indicating a similar crystal quality in these regions despite the growth temperature difference.

The Raman and PL emission spectra from the center and edge of monolayer WSe_2_ grown at 900 °C are compared in Fig. 3. The obtained PL spectra from center position is deconvoluted into three peaks: neutral exciton at ~ 1.624 eV (marked as A) [51, 52], trion at 1.60 eV (marked as A^+^) [29, 52], and an unknown emission peak (marked as D) around 1.53 eV (the detailed fitting basis are shown in Additional file 1: Figures S6–S8). Figure 3b shows the PL emission is dominated by the A^+^ in the center position. The binding energy for A^+^ is estimated to be about 24 meV, which is the energy difference between trions and neutral exciton [36]. It fits perfectly with the value of positive trion in the literature [33, 35], where the trion consists of two holes (*h*^*+*^) and an electron (*e*^*−*^). Indeed, recent studies reveal that CVD-grown WSe_2_ is usually p-type due to the formation of tungsten vacancy [27]. These results are consistent with the general rules of doping effects in semiconductors. During the power-dependent PL experiments, D emission quickly saturates (see Additional file 1: Figure S7 in the SI), suggesting that the unknown emission is actually caused by the lattice defects, as observed in other reports [24, 33, 51, 52]. In comparison, the emission from the edge does not contain this defect-related peak. Instead, the emission peak is much narrower and stronger, consisting of mainly neutral exciton peak with trion peak as a shoulder. During the power-dependent PL experiments, the FWHM of WSe_2_ on both center and edge does not change with power, indicating no signs of local heating effect (see Additional file 1: Figure S8 in the SI) [51, 60]. This defect-related emission peak becomes more obvious at low temperature (77 K), as compared in Fig. 3d. The PL spectrum at 77 K from the center region consists of three emission peaks. Through calculations, the binding energies of monolayer WSe_2_ for trion (A^+^) and defect-related emission are around 24 meV and 100 meV, respectively, which are consistent with our room temperature PL fitting results.

These results confirm the existence of the crystal defect in the CVD-grown WSe_2_ monolayer. These defects are centers for nonradioactive recombination, thus dropping the photon emission efficiency [24, 61]. Moreover, the defect density is position and growth condition dependent, leading to different emission distribution pattern in Fig. 2. Under poor growth conditions, monolayer WSe_2_ can still form. However, a large proportion of area is highly defected and contains only a small area with high crystal purity. PL spectrum and mapping provide a quick method to evaluate its crystal quality and guide the growth optimization. According to the above analysis, the monolayer WSe_2_ growth at lower growth temperature shows a weaker crystal quality, which could be due to insufficient reaction between the WO_3-*x*_ and Se gas [62, 63]. Improving the temperature could thus overcome the reaction barrier and form WSe_2_ with high crystal quality (920 °C). However, keeping increasing the temperature (940 °C) could lead to the decomposition of the formed monolayer WSe_2_ under insufficient Se gas protection [64]. Thus, the defect formation mechanism could vary at different growth temperatures, thereby leading to different emission distribution patterns. We found that the PL intensity of inner region of the triangle is the lowest. The decrease of PL intensity suggests that the crystal defects of the WSe_2_ were produced from the center of the triangle, which is consistent with previous reports [51]. In addition, the probability of lattice distortion along the armchair (see Fig. 2a) direction is larger for monolayer WSe_2_ at 900 °C. As the WSe_2_ grown from the center of the triangle to the three angle edges of the triangle, the crystal quality of WSe_2_ is getting better.

**Fig. 4:**
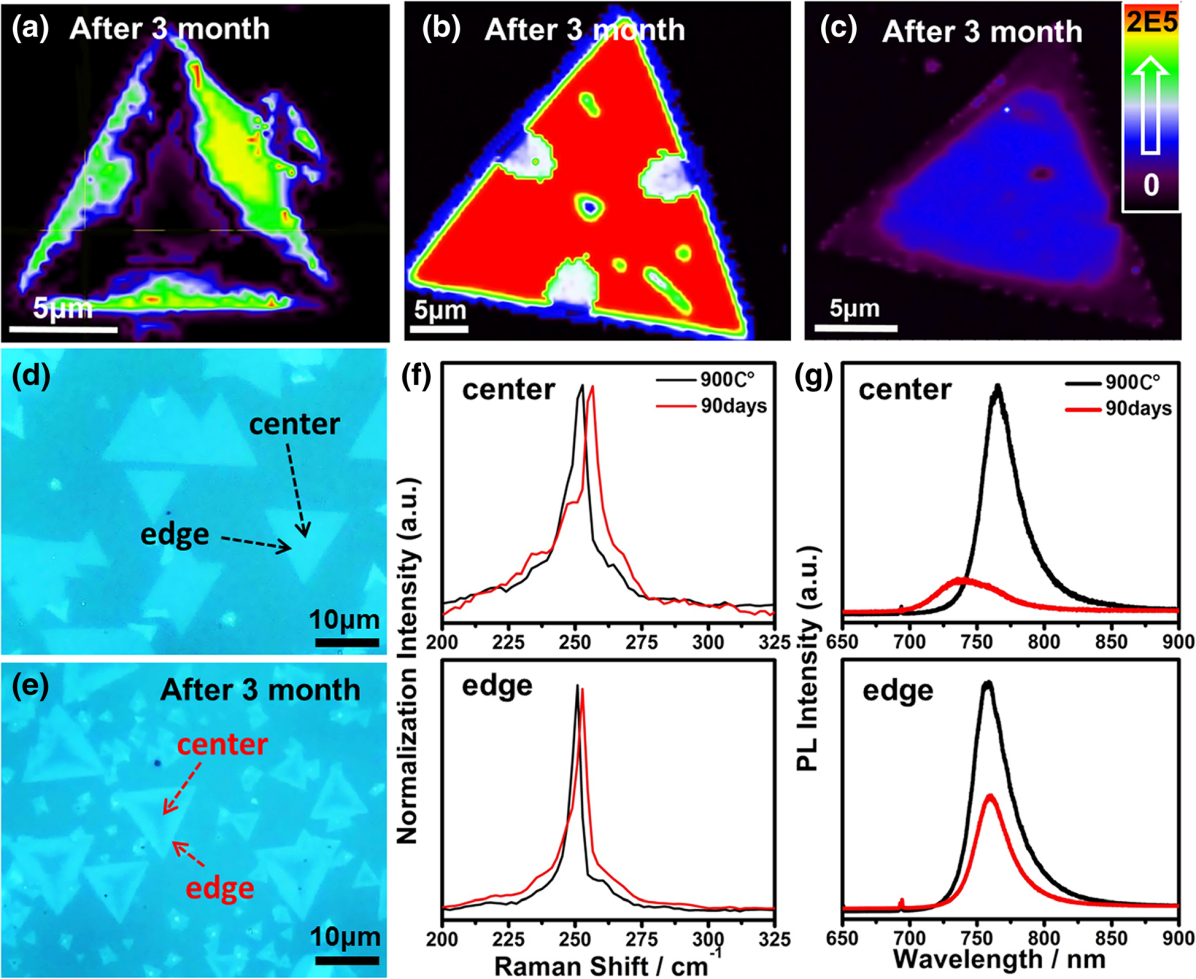
The direct correlation between crystal stability and lattice defect of of WSe_2_. PL mapping of WSe_2_ monolayer grown at **a** 900 °C, **b** 920 °C, and **c** 940 °C, respectively, after placing in the air for 90 days. Optical images of WSe_2_ grown at 900 °C **d** before and **e** after 90 days. **f** Raman and **g** PL spectra comparison from the center and the edge of the WSe_2_ sample grown at 900 °C before and after 90 days. The excitation power for PL measurements is 50 μW

**Fig. 5:**
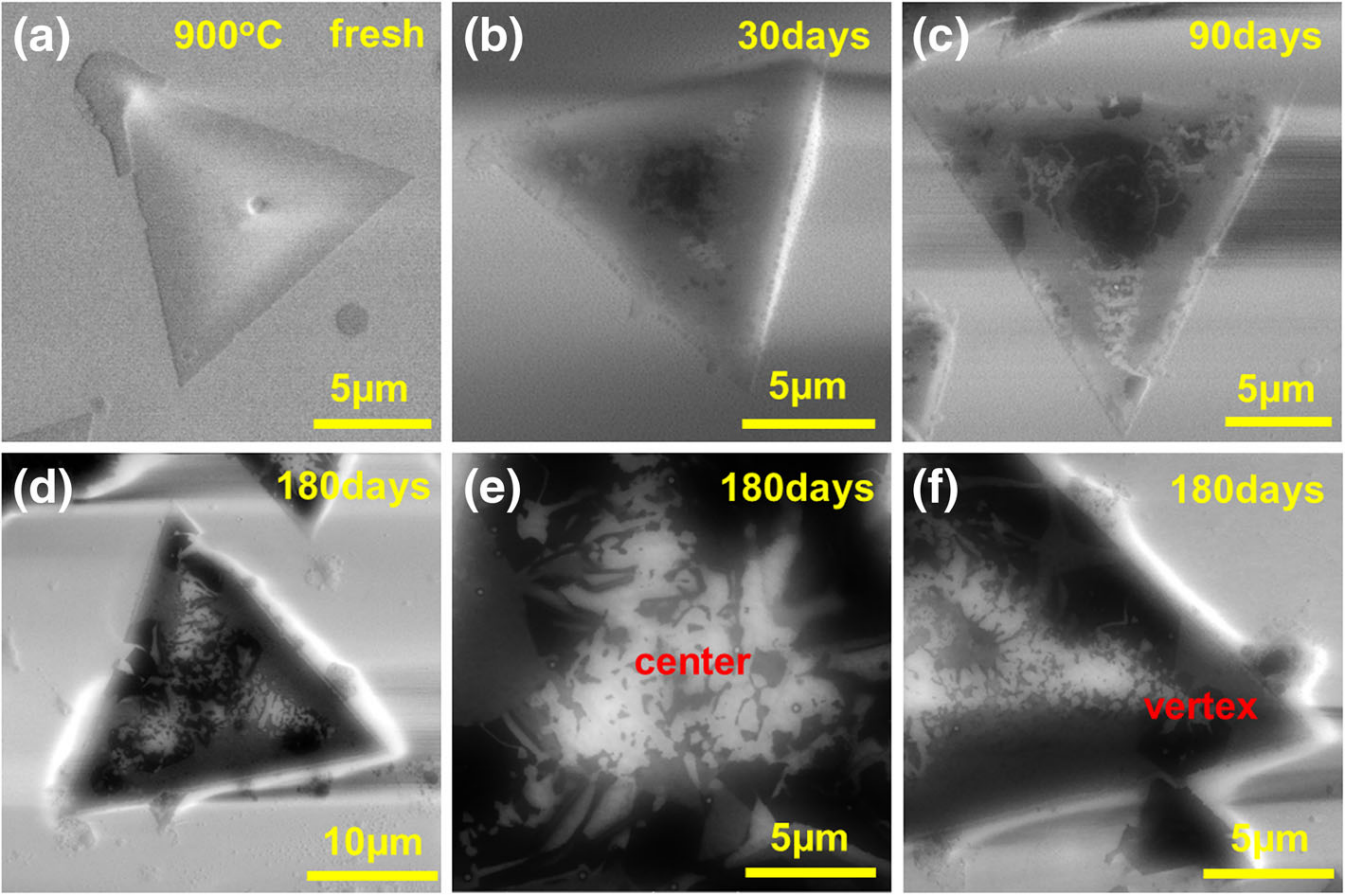
SEM images of **a** fresh monolayer WSe_2_ grown at 900 °C, placing in the air for **b** 30 days, **c** 90 days, and **d** 180 days, respectively. The enlarged view of the center and angle **f** in **d**. All the samples were stored in 25 °C. **e**, **f** Enlarged views of the center and vertex of monolayer **d**, respectively

Crystal stability is always an issue for the monolayer TMDCs crystal, and the existence of crystal defect usually makes this situation even worse. A direct relationship between the crystal defects and the decomposition of WSe_2_ is revealed in Fig. 4. After keeping the measured samples in air conditions for another 90 days, the PL emission intensity for samples grown under 900 °C and 940 °C are remarkably decreased as expected due to quick decomposition while the emission intensity distribution pattern does not change drastically. This crystal deterioration can even be observed using optical microscopy, as shown in Fig. 4d, e. The decomposed region matches perfectly with the low PL emission region in Fig. 2d. This observation suggests that the formed defects in WSe_2_ act as a center for the decomposition process, largely reducing the crystal stability in air. In contrast, WSe_2_ grown at optimal temperature with the purist crystal quality presents a much better crystal stability. The emission intensity drop is not obvious and still shows a strong PL emission. However, the emission intensity becomes inhomogeneous with weak emission at the center of the triangle edge (see more examples in Additional file 1: Figure S5). This suggests that the decomposition or crystal deterioration process in high-quality WSe_2_ begins from the center of the triangle edge. The PL and Raman spectra of WSe_2_ grown at 900 °C before and after 90 days are compared in Fig. 4f, g. The E^1^_2g_ vibration mode of the center region is red-shifted by ~ 3.7 cm^−1^ while this shift is only ~ 1.9 cm^−1^ at the edge region. As discussed in Fig. 1, the results show that the crystal quality deteriorates faster in the region with a higher density of lattice defects. The existence of lattice defects would lower the energy barrier for WSe_2_ decomposition and accelerate the decomposition process. The region with a higher defect density can easily combine with O and OH, deteriorating its lattice stability [25]. This process then gradually propagates throughout the entire monolayer WSe_2_. This lattice evolution process matches perfectly with our aging experiment processes (see Figs. 4e and 5). Consequently, WSe_2_ grown at 900 °C starts to decompose from the center region. In comparison, WSe_2_ grown at 920 °C decomposes more slowly due to a better crystal quality. And the decomposition initiates from the more chemically active regions, such as edges and grain boundaries [65], as has been demonstrated in Fig. 4b.

The PL emission in Fig. 4g shows a similar trend. Compared with the data measured 90 days before, the PL peak position and emission intensity of the center region are blue-shifted by ~ 60 meV and decreased 7 times, respectively. Moreover, the FWHM is broadened by ~ 17 meV. In contrast, the PL peak position and FWHM of the edge are nearly the same and the emission intensity only drops to half of the intensity measured at 90 days before. Using the same approach, we found that the crystal deterioration process in monolayer WSe_2_ grown at 940 °C shows the same mechanism: the higher the crystal quality, the slower is the decomposition.

In order to better understand the aging process, the morphology evolution of monolayer WSe_2_ grown at 900 °C with time is shown in Fig. 5. The aged region starts from the center of the triangle (see Fig. 5b). As the aging time increases, WSe_2_ decomposes gradually from the center to the vertex of the triangle as shown in Fig. 5c. After 180 days, WSe_2_ at the center of the triangle and the three angular positions have been substantially decomposed completely. At this time, the PL in the center and triangle has quenched. Raman scattering in these decomposed areas shows no signal of vibration mode of WSe_2_, confirming the complete decomposition of WSe_2_ crystal. The aging study of a single layer of WSe_2_ grown at 900 °C further demonstrates that the location of the decomposition agrees very well with our previously measured PL mapping results. According to the above discussions, the critical factor affecting the stability of WSe_2_ is the unwanted defect formation during the CVD growth. PL and Raman spectrum provides an easy approach to quickly examine the crystal quality to guide the growth optimization towards 2D layer with the purest crystal quality.

## Conclusion

In summary, we study the role of growth temperature on crystal defect formation and crystal stability of monolayer WSe_2_ on a sapphire substrate. PL and Raman spectroscopy techniques are applied to quickly identify the crystal quality, stability, and defect distribution of as-grown monolayer WSe_2_ at different conditions. Through this characterization approach, the optimal growth temperature for monolayer WSe_2_ is obtained at 920 °C. Either reducing or increasing the growth temperature leads to the formation of a higher defect density. At lower growth temperature, the defect formation is probably due to the unfully decomposed WO_3-*x*_ precursor. The defects start to form at the nucleus center and then proceed along the armchair direction of the crystal, forming an inner triangular shape with a high density of defects and lower PL emission intensity. Above the optimal growth temperature, the defect distribution shows another pattern and starts from the edge, probably due to the decomposition of WSe_2_ at such a high temperature. PL emission shows that photon emission in the defected region is dominated by trions while neutral exciton emission is prominent in the WSe_2_ monolayer with better crystal quality. The aging experiment further proved that the region with a higher defect density can easily combine with O and OH, deteriorating its lattice stability. These results offer insights into the optimum synthesis of various 2D materials and the potential applications in the field of optoelectronics.

## Additional file

Additional file 1: **Figure S1.** More optical images of WSe_2_ samples grown on sapphire substrates: (a) 860 ^o^C, (b) 880 ^o^C, (c) 900 ^o^C, (d) 920 ^o^C and (e) 940 ^o^C. **Figure S2.** Optical microscopy images of WSe_2_ grown for (a) 4 min, (b) 7 min, (c) 10 min and (d) 20 min. The growth temperatures are 920 ^o^C for all cases. **Figure S3.** Raman spectra of five different monolayer WSe_2_ samples grown at (a) 860 ^o^C, (b) 880 ^o^C, (c) 900 ^o^C, (d) 920 ^o^C and (e) 940 ^o^C, respectively. **Figure S4.** Raman spectra of 5 different monolayer WSe_2_ samples grown at (a) 860 ^o^C, (b) 880 ^o^C, (c) 900 ^o^C, (d) 920 ^o^C and (e) 940 ^o^C, respectively. **Figure S5.** More PL integral intensity mapping of WSe_2_ monolayer: (a) 900 ^o^C, (b) 920 ^o^C and (c) 940 ^o^C, respectively. (d) PL intensity mapping of WSe_2_ grown at 920 ^o^C after placing in the air for another 90 days. **Figure S6.** Deconvoluted spectra obtained with excitation laser power levels of 5 μW, 10 μW, 50 μW, 100 μW and 500 μW, respectively, corresponding to the positions in the center region. **Figure S7.** Deconvoluted spectra obtained with excitation laser power levels of 5 μW, 10 μW, 50 μW, 100 μW and 500 μW, respectively, corresponding to the positions in the edge region. **Figure S8.** The deconvoluted PL peak position (a) and FWHM (b) of neutral exciton (A), trion (A^+^), and defects (D) as a function of laser power at the center and edge regions, respectively. The excitation power is 50 μW. (DOCX 5032 kb)

## Data Availability

All data are fully available without restriction.

## Abbreviations

2D: two-dimensional
AFM: Atomic force microscope
CVD: Chemical vapor deposition
FWHM: Full width at half maximum
PL: Photoluminescence
sccm: standard-state cubic centimeter per minute
SEM: Scanning electron microscope
STM: Scanning tunneling microscopy
TEM: Transmission electron microscopy
TMDCs: Transition metal dichalcogenides
