# Supplementary material for: Quick Optical Identification of the Defect Formation in Monolayer WSe2 for Growth Optimization
Source: Nanoscale Res Lett. 2019 Aug 14;14:274. doi: 10.1186/s11671-019-3110-z (PMC6692796; doi:10.1186/s11671-019-3110-z)
Supplement: Supplementary file 1 — Figure S1. More optical images of WSe2 samples grown on sapphire substrates: (a) 860 oC, (b) 880 oC, (c) 900 oC, (d) 920 oC and (e) 940 oC. Figure S2. Optical microscopy images of WSe2 grown for (a) 4 min, (b) 7 min, (c) 10 min and (d) 20 min. The growth temperatures are 920 oC for all cases. Figure S3. Raman spectra of five different monolayer WSe2 samples grown at (a) 860 oC, (b) 880 oC, (c) 900 oC, (d) 920 oC and (e) 940 oC, respectively. Figure S4. Raman spectra of 5 different monolayer WSe2 samples grown at (a) 860 oC, (b) 880 oC, (c) 900 oC, (d) 920 oC and (e) 940 oC, respectively. Figure S5. More PL integral intensity mapping of WSe2 monolayer: (a) 900 oC, (b) 920 oC and (c) 940 oC, respectively. (d) PL intensity mapping of WSe2 grown at 920 oC after placing in the air for another 90 days. Figure S6. Deconvoluted spectra obtained with excitation laser power levels of 5 μW, 10 μW, 50 μW, 100 μW and 500 μW, respectively, corresponding to the positions in the center region. Figure S7. Deconvoluted spectra obtained with excitation laser power levels of 5 μW, 10 μW, 50 μW, 100 μW and 500 μW, respectively, corresponding to the positions in the edge region. Figure S8. The deconvoluted PL peak position (a) and FWHM (b) of neutral exciton (A), trion (A+), and defects (D) as a function of laser power at the center and edge regions, respectively. The excitation power is 50 μW. (DOCX 5032 kb) [file 11671_2019_3110_MOESM1_ESM.docx]

**Supporting Information:**

**Quick optical identification of the defect formation in monolayer WSe_2_ for growth optimization**

**Long Fang^1^, Haitao Chen^2^, Xiaoming Yuan^1,*^ Han Huang^1^, Gen Chen^3^, Lin Li^1^, Junnan Ding^1^, Jun He^1^, Shaohua Tao^1,*^**

*^1^Hunan Key Laboratory of Super Micro-structure and Ultrafast Process, School of Physics and Electronics, Central South University, Changsha 410083, China*

*^2^College of Advanced Interdisciplinary Studies, National University of Defense Technology, Changsha 410083, China*

*^3^School of Materials Science and Engineering, Central South University, Changsha 410083, China*

*** Corresponding authors: [xiaoming.yuan@csu.edu.cn](mailto:xiaoming.yuan@csu.edu.cn); [eshtao@csu.edu.cn](mailto:eshtao@csu.edu.cn)

**S5-S7**：The emission peak at center can be deconvoluted to three peaks: neutral exciton (marked A, 1.624 eV), trion exciton (1.600 eV) and unknown emission (1.530 eV). The binding energies for trion exciton and unknown emission were estimated to be about 24.5 meV and 100 meV, respectively. Hence, marking trion exciton as A^+^ for two reasons: (1) the binding energy of positive trion obtained by fitting in the experiment is perfectly consistent with the literature,[[1](#_ENREF_1)] so the trion is comprise by binding two holes (h^+^) and an electron(e^-^); (2) the latest study reveals that the tungsten vacancy is the cause of hole (p-type) doping,[[2](#_ENREF_2)] more holes than electrons also makes it possible for trion (A^+^) formation. The low energy peak (1.530 eV) is usually ascribed to the point defect (marked D, 1.530eV) which is consistent with some recent reports.[[3](#_ENREF_3)]


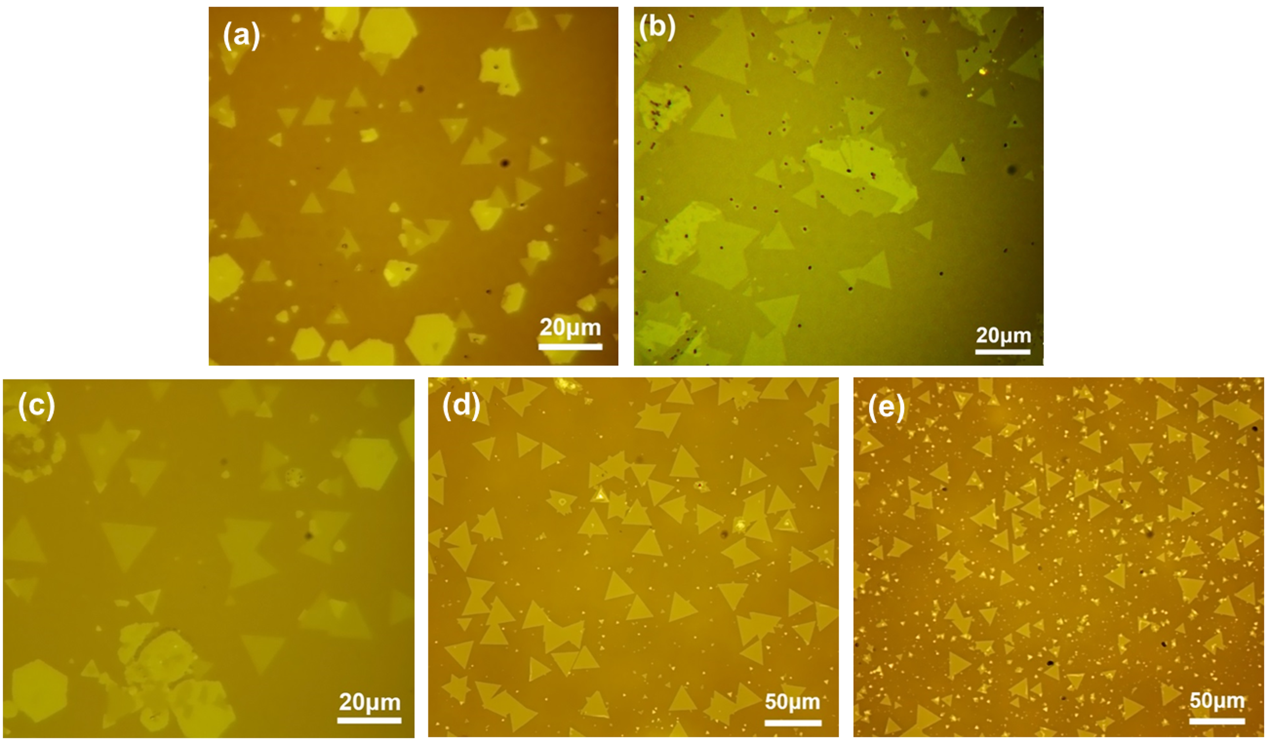


**Figure S1** More optical images of WSe_2_ samples grown on sapphire substrates: (a) 860 ^o^C, (b) 880 ^o^C, (c) 900 ^o^C, (d) 920 ^o^C and (e) 940 ^o^C.


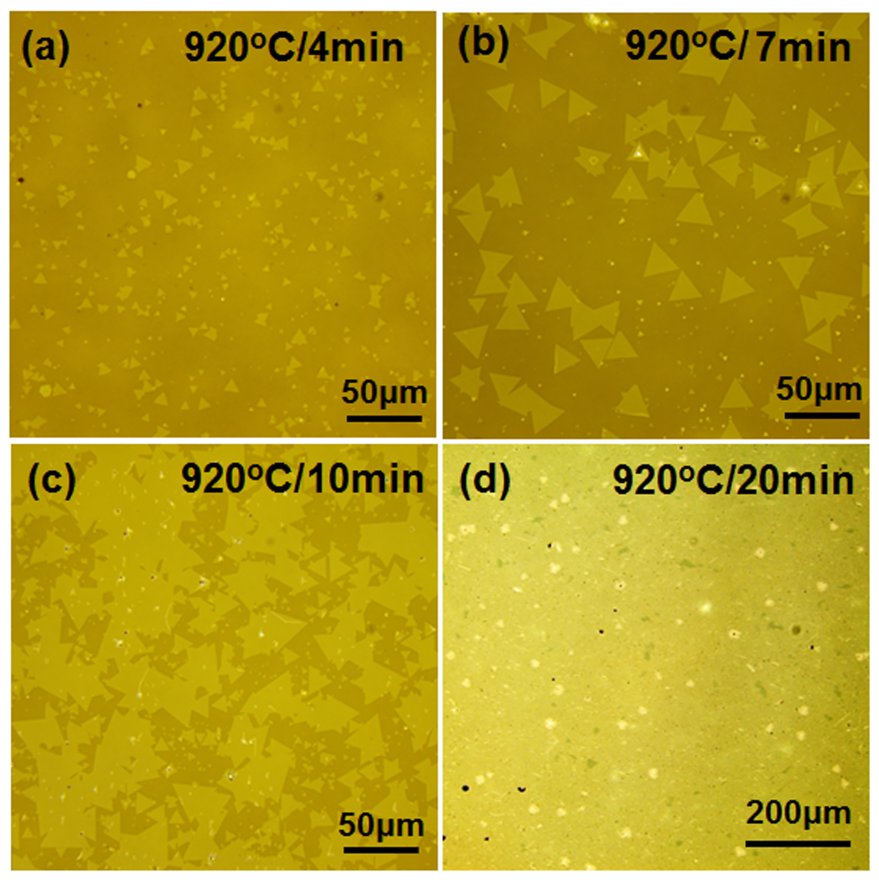


**Figure S2** Optical microscopy images of WSe_2_ grown for (a) 4 min, (b) 7 min, (c) 10min and (d) 20min. The growth temperatures are 920 ^o^C for all cases.


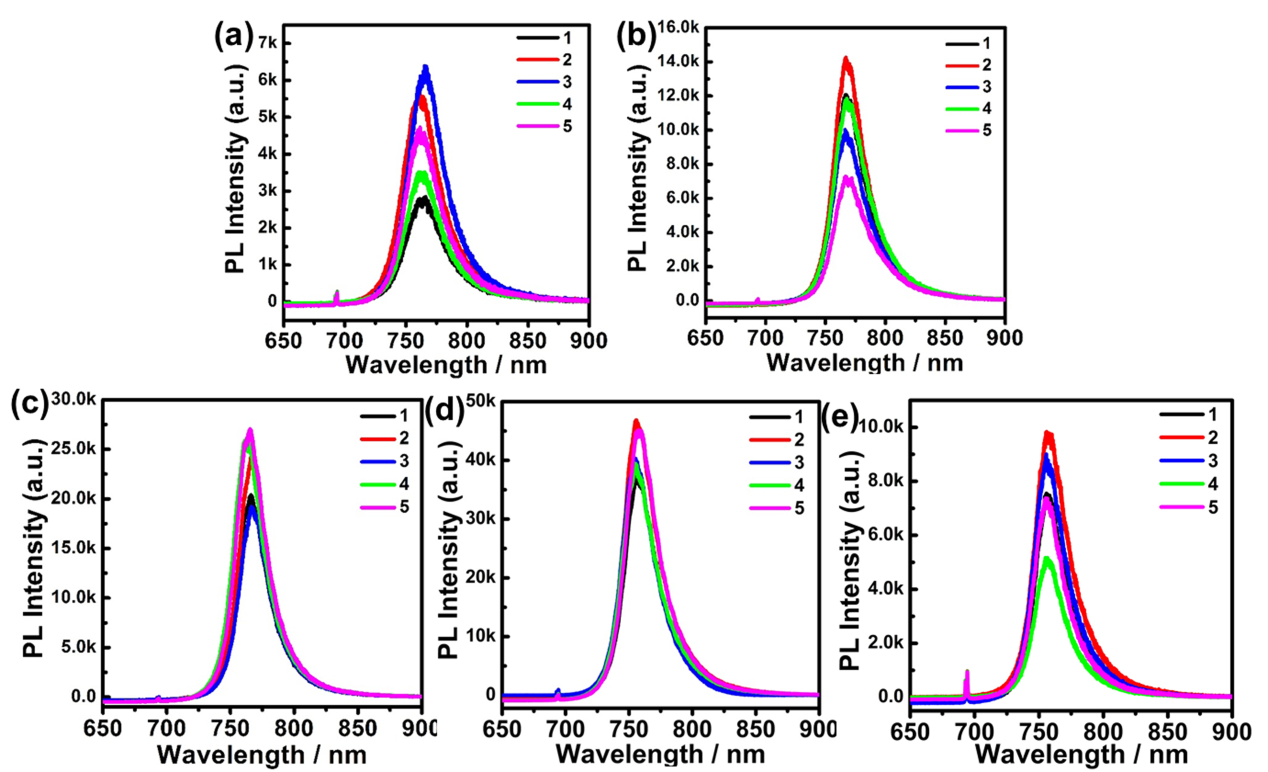


**Figure S3** Raman spectra of 5 different monolayer WSe_2_ samples grown at (a) 860 ^o^C, (b) 880 ^o^C, (c) 900 ^o^C, (d) 920 ^o^C and (e) 940 ^o^C, respectively.


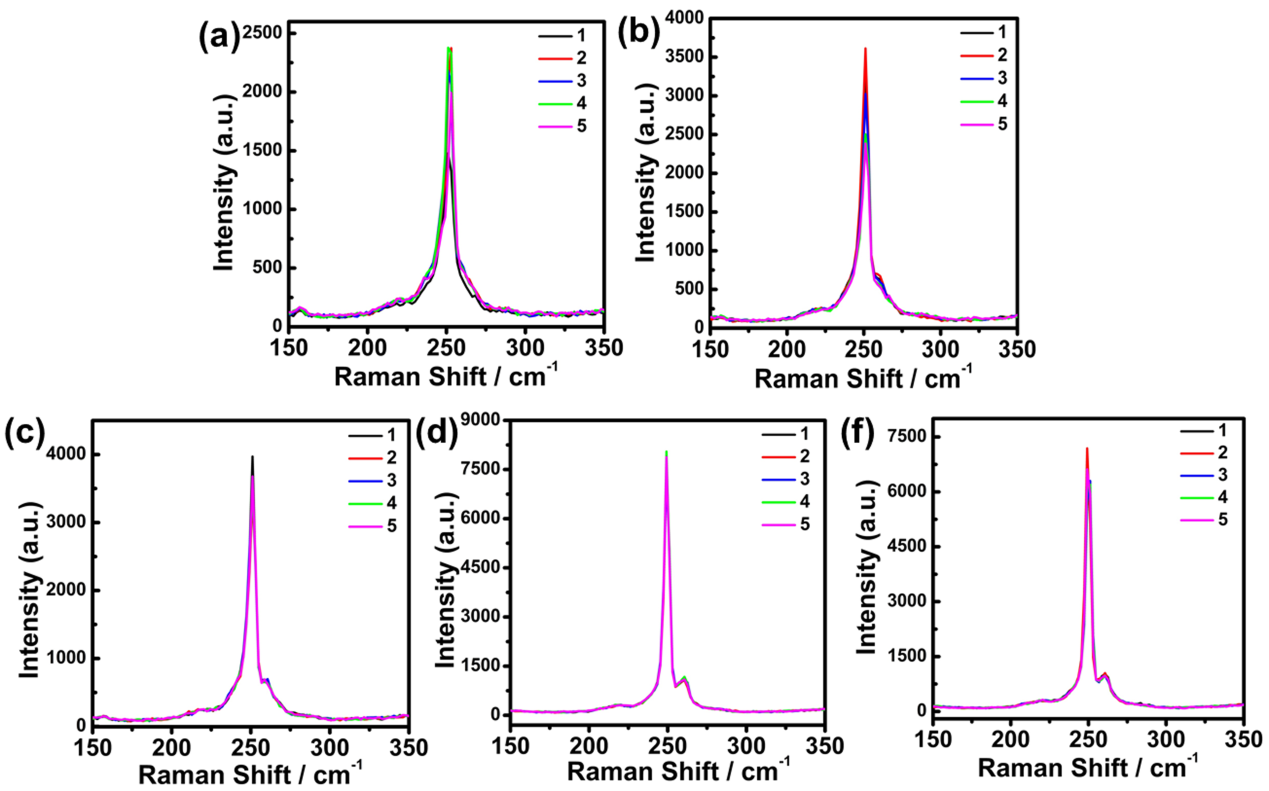


**Figure S4** Raman spectra of 5 different monolayer WSe_2_ samples grown at (a) 860 ^o^C, (b) 880 ^o^C, (c) 900 ^o^C, (d) 920 ^o^C and (e) 940 ^o^C, respectively.


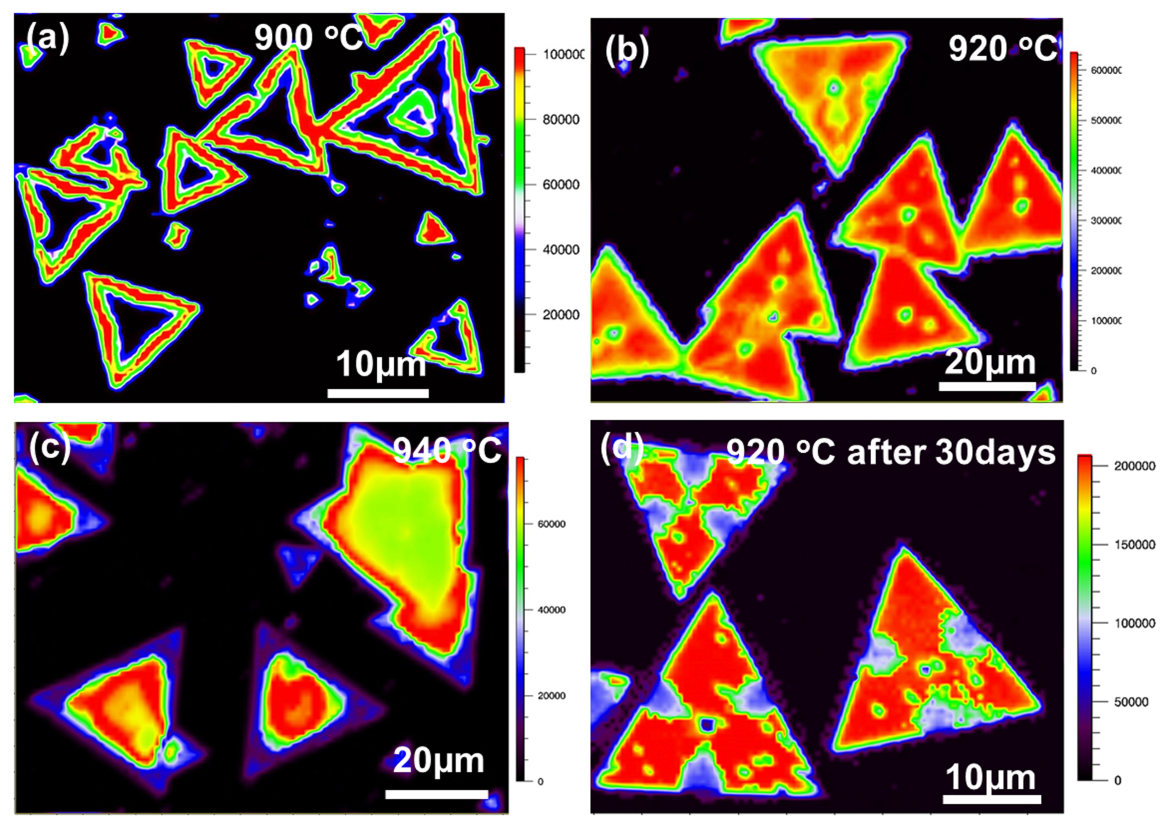


**Figure S5** More PL integral intensity mapping of WSe_2_ monolayer: (a) 900 ^o^C, (b) 920 ^o^C and (c) 940 ^o^C, respectively. (d) PL intensity mapping of WSe_2_ grown at 920 ^o^C after placing in the air for another 90 days.


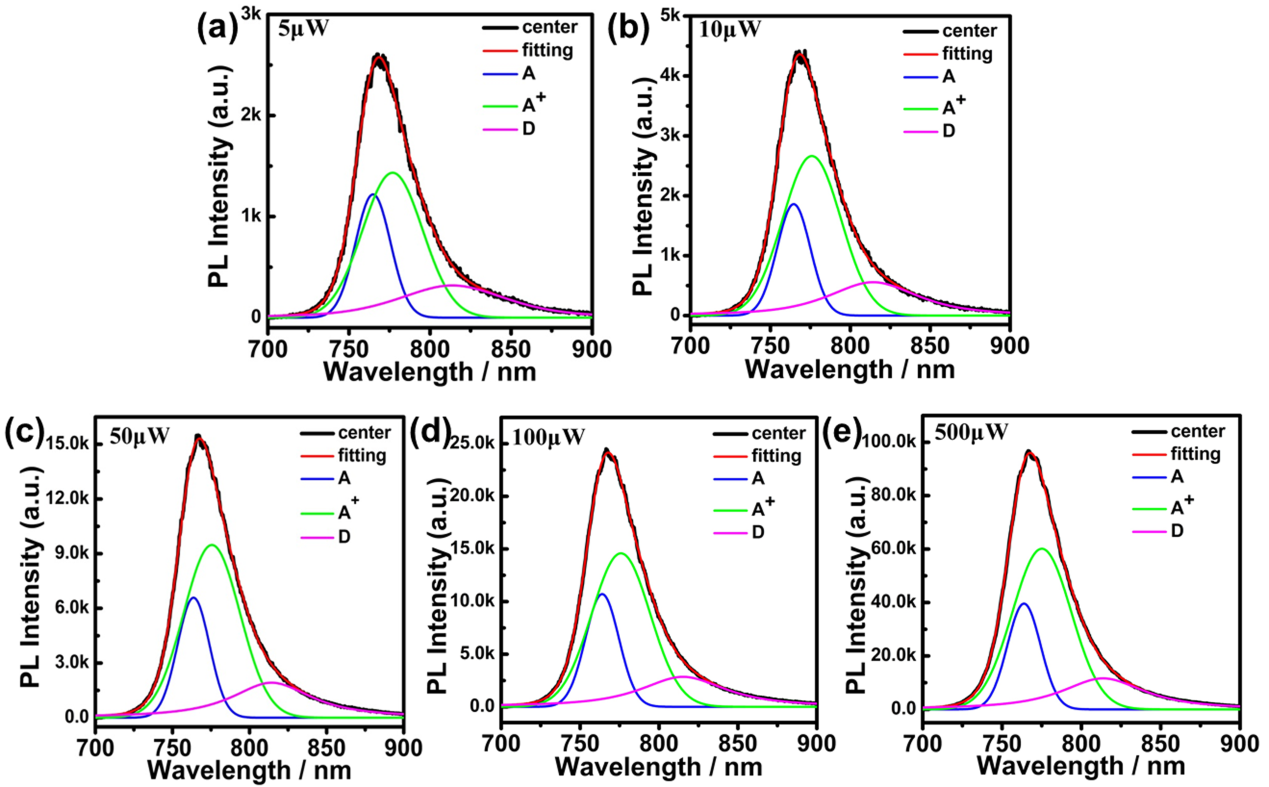


**Figure S6** Deconvoluted spectra obtained with excitation laser power levels of 5μW, 10μw, 50μW, 100μW and 500μW, respectively, corresponding to the positions in the center region.


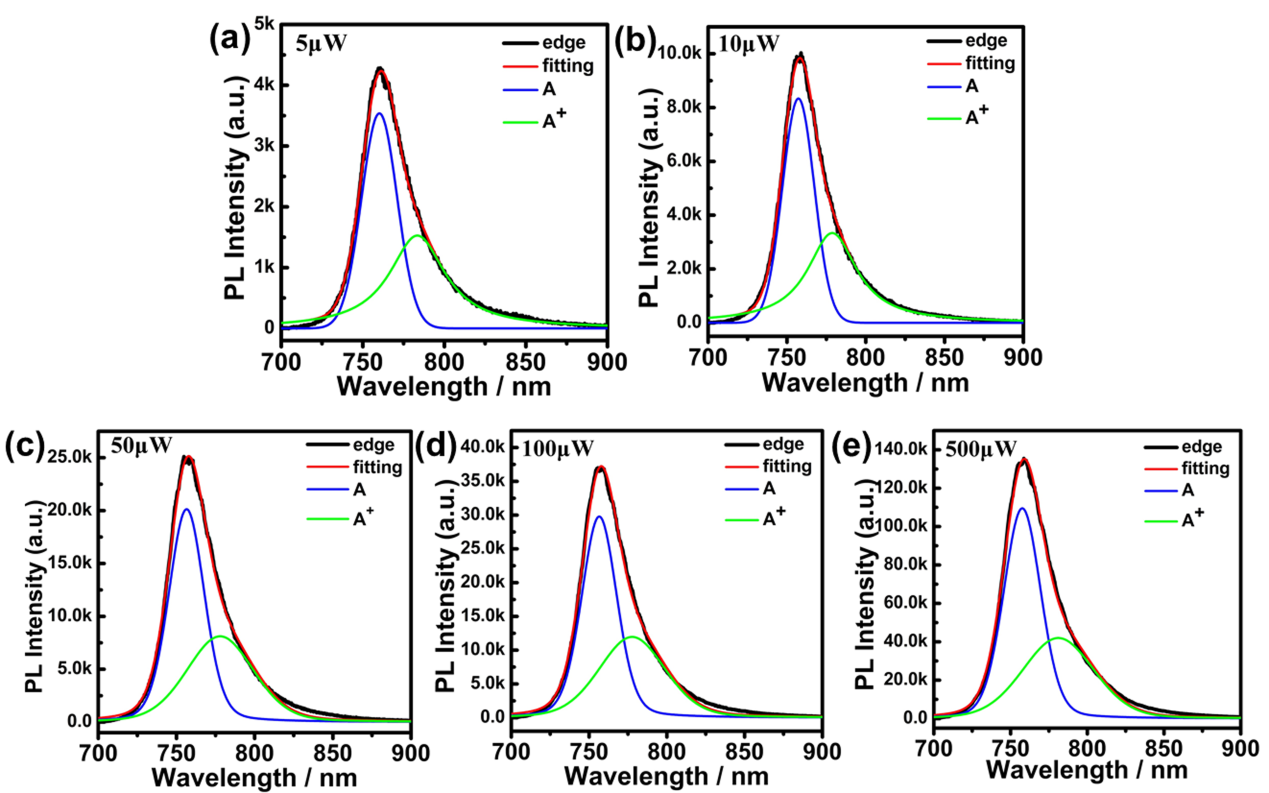


**Figure S7** Deconvoluted spectra obtained with excitation laser power levels of 5μW, 10μw, 50μW, 100μW and 500μW, respectively, corresponding to the positions in the edge region.


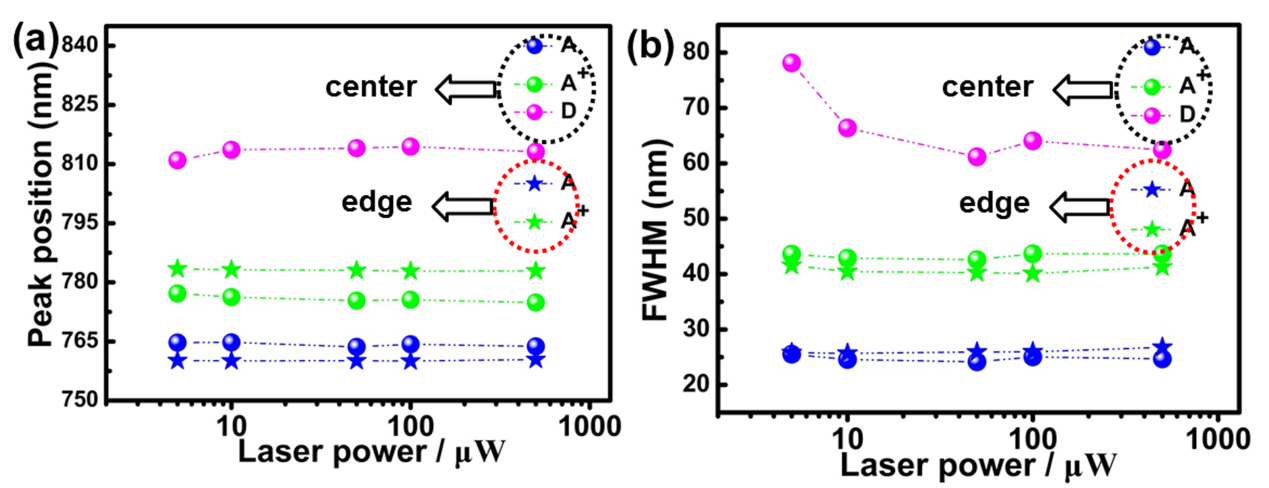


**Figure S8** The deconvoluted PL peak position (a) and FWHM (b) of neutral exciton (A), trion (A^+^), and defects (D) as a function of laser power at the center and edge regions, respectively. The excitation power is 50μW.

References

1. Jones AM, Yu HY, Ghimire NJ, Wu SF, Aivazian G, Ross JS, Zhao B, Yan JQ, Mandrus DG, Xiao D, Yao W, Xu XD (2013) Optical generation of excitonic valley coherence in monolayer WSe_2_. Nat Nanotechnol 8:634-638.

2. Zhang S, Wang CG, Li MY, Huang D, Li LJ, Ji W, Wu SW (2017) Defect Structure of Localized Excitons in a WSe_2_ Monolayer. Phys Rev Lett 119:046101.

3. Li TS, Li ML, Lin Y, Cai HB, Wu YM, Ding HY, Zhao SW, Pan N, Wang XP (2018) Probing Exciton Complexes and Charge Distribution in Inkslab-Like WSe_2_ Homojunction. ACS Nano 12:4959-4967.
